# Supplementary material for: Progression of Chronic Kidney Disease and All-Cause Mortality in Patients with Tricuspid Regurgitation
Source: Diseases. 2022 Mar 16;10(1):16. doi: 10.3390/diseases10010016 (PMC8946925; doi:10.3390/diseases10010016)
Supplement: Supplementary file 1 [file diseases-10-00016-s001.zip › diseases-1621293-supplementary.pdf]

Table S1 – Literature search for "tricuspid regurgitation" and "chronic kidney disease" and "mortality"

Reference	Study Design	Patients	Follow-up time	Primary endpoint	Patients	Incidence CKD ≥3	Independent predictors of CKD	Independent predictors of CKD progression	Independent predictors of mortality	Further results
Ingraham BS et al. Heart 2019	Retrospective observational cohort study	N=87 patients	331 days	-	patients with severe TR considered for intervention	64%	-	-	-	30-day and 1-year survival were 100% and 76% / Patients with greater EROA, better RV function and more severe symptoms were more likely to receive intervention.
Dietz MF et al. Circulation 2019	Retrospective cohort study	N=1,292 patients	34 months	all-cause mortality	Patients with significant secondary TR	-	-	-	LVEF (OR 1.44), RV systolic pressure (OR 1.016), Baseline creatinine (OR 1.004)	RV remodelling varies significantly: RV dilation and systolic dysfunction were present in 43% of patients,
Mehr M et al. Jacc Cardiovasc Interv. 2019	Multicentre international TriValve (Transcathete r Tricuspid Valve Therapies) registry	N=249 patients	1-year	Clinical and echocardi o-graphic outcomes	severe TR treated with edge-to-edge repair	6.8% (on dialysis)	-	-	procedural failure OR 2.12, worsening kidney function OR 1.25 per 10ml/min eGFR, absence of sinus rhythm OR 4.40.	All cause mortality at 1-year follow-up 20% Predictors of procedural failure: absence of central and/or anteroseptal TR jet location, larger coaptation gap size, larger tricuspid tenting area, and larger EROA
Benfari G et al. Circulation 2019	Retrospective cohort study	N=13.02 6 patients		5-year mortality	Mayo Clinic patients from 2003 to 2011 diagnosed with heart failure stage B-C	36%	-	-	higher TR degree OR 1.09 for mild TR; 1.21 for moderate and 1.57 for severe TR	TR was detected in 88% and was independently associated with impaired kidney function.
Bannehr M et al. J Cardiol 2019	Retrospective observational cohort study	N=213 patients	1458 days	Long-term survival	consecutive patients considered PMVR	65%	-	-	post-procedural TR grade (OR 2.06), impaired LV function (OR3.1) CKD (OR4.0 – eGFR<30ml/min/1.73)	-
Omote K et al. J Card Fail 2019	Multicentre Registry	N=469 patients	748 days	all-cause death	consecutive hospitalized patients with	52.5%	-	-	elevated TRPG as independent risk factor for mortality (OR1.02) -	Admission TRPG correlated with estimated pulmonary

					HFpEF + measurement of TRPG				adjustment of renal function	capillary wedge pressure and left atrial dimension
Murana G et al. Interact Cardiovasc Thorac Surg 2018	Retrospective observational cohort study	N=318 patients	5.6 years	Long-term survival	Patients with mitral valve replacement	9.1% (creatinine >200 µmol/L)	-	-	Hypertension (OR 1.91), COPD (OR 2.91) and CKD (OR 5.27)	overall hospital mortality rate was 4.4%
Agricola E et al. J Cardiovasc Med (Hagerstown). 2017	Retrospective observational cohort study	N=413 patients	36 months	Long-term survival	Chronic heart failure with LVEF <50%	48%	Age (OR 1.1); Diabetes (OR 1.2); AF (OR 1.8) Reduced LVEF (OR 0.97); NYHA III-IV (OR 1.3)	-	CKD (OR 2.3), Moderate/severe MR (OR 1.6), NT-proBNP (OR 1.3), NYHA III-IV (OR 2.7), AF (OR 1.4) ICD (OR 0.18), TAPSE (<16 mm) (OR 1.3), Estimated RAP (OR 1.4)	combination of significant functional TR + right ventricular dysfunction, but not functional TR and right ventricular dysfunction alone, is independently associated with renal dysfunction.
Omrani H et al. Med Arch 2016	Observational cross-sectional study	N=150 patients	1 year	Echocardio-graphic findings before first and after 1-year HD	Advanced kidney disease with upcoming HD	100%	-	-	-	prevalence of all echocardiographic findings was more after the first dialysis compared with before the first dialysis in diabetic patients (P<0.05)
Gluba-Brzozka, A., et al. Lipids Health Dis 2016	Observational cohort study	N=104 patients	-	athero-sclerosis in CKD	patients hospitalized with CKD (N=80 patients (CKD I-IV), N=24 healthy volunteers)	57,7%	-	-	-	tricuspid insufficiency (27.3 % in CKD I/II vs. 71.4% in CKD V, p = 0.016)
Bushyhead D et al. Liver Transpl 2016	Retrospective cohort study	N=397 patients	5 years	Long-term survival	liver transplant recipients	-	Female (OR 0.53), Hyper-tension (OR 2.33), Serum Creatinine at transplant (OR 2.38), LVEF ≥65% (OR 1.75)	-	TR graded greater than mild was associated with significantly increased posttransplant mortality (OR 1.68); smoking (OR 1.66)	pretransplant echocardiography may be used as a tool to risk-stratify patients for posttransplant outcomes
Taramasso M et al. J Card Surg	Observational cohort study	N=61 patients	3.2 years	all-cause death	patients with aortic valve replacement	13%	-	-	TR >= 3+ at follow-up (OR 10.58), preoperative NYHA	preoperative untreated TR 2/4R improves or remains stable in the

2016									class III– IV (OR 8.01)	majority of patients.
Navaneethan SD et al. J Am Soc Nephrol. 2016	Multicentre cohort study	N=3,939 patients	4.1 years	Cardio-vascular events	eGFRs of 20–70 ml/min per 1.73 m2	100%	-	-	Pulmonary hypertension (OR 1.38)	Pulmonary hypertension and higher TRV and PASP are associated with adverse outcomes in CKD
Busch C, et al. Eur J Cardiothorac Surg. 2015	Retrospective observational cohort study	N=97 patients	3.1 years	Long-term survival	Patients undergoing surgery for constrictive pericarditis	27.2%	-	-	LVEF (35%< LVEF <55%) (OR 3.6), right ventricular dilatation (OR 3.5) long-term mortality: CAD (OR 6.4) CKD (OR 1.8), COPD (OR 4.2)	Surgery for constrictive pericarditis is associated with a significant risk based on the poor preoperative patient status
Fernando M et al. J Thorac Cardiovasc Surg. 2014	Multicentre Registry (Cardiothoracic surgical databases in 3 hospitals)	N=545 patients	-	Long-term survival	Mixed cardiac surgery patients with and without valve surgery and with CKD	-	-	eGFR <20 mL/min (OR 4.6) as the only predictor for starting permanent dialysis within 6 months	age (OR 1.4 per decade), emergency surgery (OR 7.0), redo surgery (OR 3.8), left ventricular impairment (moderate, OR 2.7; severe, OR 4.4)	emergency surgery (OR 2.7), tricuspid valve surgery (OR, 4.4), eGFR < 20 mL/min (OR, 3.8), independent predictors of early postoperative dialysis
Ohno Y et al. Heart J Cardiovasc Imaging 2014	Observational cohort study	N=146 patients	20 months	freedom from death, surgery for mitral valve dysfunction, or grade ≥ 3+ MR at 12-month FU	MitraClip recipients -none/mild TR (N=99) -moderate/severe TR (N=47)	47.5% (TR 0/1) 57.4% (TR 2/3)	-	-	Combined Endpoint: mortality and re-hospitalization: baseline moderate/ severe TR (OR: 2.67), CKD (OR: 3.16)	MitraClip led to improvement in MR, TR, and NYHA functional class in patients with baseline moderate/severe TR
Ro S et al. J Thorac Cardiovasc Surg 2013	Retrospective observational cohort study	N=959 patients	64.8 months	all-cause mortality	patients with mild-to-moderate TR who underwent mitral valve surgery with or without concomitant TV repair.	<1%	-	-	Composite endpoint: Age (OR 1.04), AF (OR 1.66), Diabetes (OR 2.08), CKD (OR 4.28), LVEF (OR 0.76) Redo surgery (OR 2.52)	-
Langanay T et al. J Heart	Retrospective observational	N= 442 patients	NA	all-cause mortality	aged > or =80 years with aortic	NA	-	-	AI (30%, p <0.004), NYHA class IV (20.5%,	Mortality was also increased in case of

Valve Dis. 2006	cohort study		valve replacement		p < 0.001), left and right heart failure (11.5% and 19.4%, p <0.02), CKD (18.5%, p <0.04), emergency (37.5%, p <0.001), LVEF (21.1%, p <0.004), and redo surgery (35.3%, p <0.001)	associated coronary revascularization (11.6%), mitral or tricuspid surgery (20%) and ascending aorta procedure (25%).

TR, tricuspid regurgitation; EROA, effective regurgitant orifice area; RV, right ventricular; LV, left ventricular; LVEF, left ventricular ejection fraction; OR, odds ratio; eGFR, estimated glomerular filtration rate; PMVR, percutaneous mitral valve repair; CKD, chronic kidney disease; HFpEF, Heart Failure with preserved Ejection Fraction; TRPG, tricuspid regurgitation pressure gradient; COPD, chronic obstructive pulmonary disease; AF, atrial fibrillation; MR, mitral regurgitation; ICD, implantable cardioverter defibrillator; TAPSE, tricuspid annular plain systolic excursion; RAP, right atrial pressure; HD, haemodialysis; TRV, tricuspid regurgitant velocity; PASP, Pulmonary arterial systolic pressure; CAD, coronary artery disease; FU, follow-up; AI, aortic insufficiency

Table S2. Research Checklist - STROBE 2007 Statement.

Section/Topic	Item #	Recommendation	Reported on page #
**Title and abstract**	1	(*a*) Indicate the study's design with a commonly used term in the title or the abstract	1
		(*b*) Provide in the abstract an informative and balanced summary of what was done and what was found	1
		**Introduction**	
Background/rationale	2	Explain the scientific background and rationale for the investigation being reported	2
Objectives	3	State specific objectives, including any prespecified hypotheses	2
		**Methods**	
Study design	4	Present key elements of study design early in the paper	2
Setting	5	Describe the setting, locations, and relevant dates, including periods of recruitment, exposure, follow-up, and data collection	3
Participants	6	(*a*) Give the eligibility criteria, and the sources and methods of selection of participants. Describe methods of follow-up	3
		(*b*) For matched studies, give matching criteria and number of exposed and unexposed	NA
Variables	7	Clearly define all outcomes, exposures, predictors, potential confounders, and effect modifiers. Give diagnostic criteria, if applicable	3
Data sources/ measurement	8*	For each variable of interest, give sources of data and details of methods of assessment (measurement). Describe comparability of assessment methods if there is more than one group	3
Bias	9	Describe any efforts to address potential sources of bias	3
Study size	10	Explain how the study size was arrived at	flow chart
Quantitative variables	11	Explain how quantitative variables were handled in the analyses. If applicable, describe which groupings were chosen and why	3
Statistical methods	12	(*a*) Describe all statistical methods, including those used to control for confounding	4
		(*b*) Describe any methods used to examine subgroups and interactions	4
		(*c*) Explain how missing data were addressed	4
		(*d*) If applicable, explain how loss to follow-up was addressed	4
		(*e*) Describe any sensitivity analyses	NA
		**Results**	
Participants	13*	(a) Report numbers of individuals at each stage of study-e.g., numbers potentially eligible, examined for eligibility, confirmed eligible, included in the study, completing follow-up, and analysed	flow chart
		(b) Give reasons for non-participation at each stage	flow chart
		(c) Consider use of a flow diagram	flow chart
Descriptive data	14*	(a) Give characteristics of study participants (e.g., demographic, clinical, social) and information on exposures and potential confounders	5-7
		(b) Indicate number of participants with missing data for each variable of interest	18-24 (Table subheadings)
		(c) Summarise follow-up time (e.g., average and total amount)	5-7
Outcome data	15*	Report numbers of outcome events or summary measures over time	7
Main results	16	(*a*) Give unadjusted estimates and, if applicable, confounder-adjusted estimates and their precision (eg, 95% confidence interval). Make clear which confounders were adjusted for and why they were included	5-7
		(*b*) Report category boundaries when continuous variables were categorized	5-7
		(*c*) If relevant, consider translating estimates of relative risk into absolute risk for a meaningful time period	8
Other analyses	17	Report other analyses done—e.g., analyses of subgroups and interactions, and sensitivity analyses	9
**Discussion**			
Key results	18	Summarise key results with reference to study objectives	10

**Limitations**			
Interpretation	20	Give a cautious overall interpretation of results considering objectives, limitations, multiplicity of analyses, results from similar studies, and other relevant evidence	10
Generalisability	21	Discuss the generalisability (external validity) of the study results	11
**Other information**			
Funding	22	Give the source of funding and the role of the funders for the present study and, if applicable, for the original study on which the present article is based	11

Note: An Explanation and Elaboration article discusses each checklist item and gives methodological background and published examples of transparent reporting. The STROBE checklist is best used in conjunction with this article (freely available on the Web sites of PLoS Medicine at http://www.plosmedicine.org/, Annals of Internal Medicine at http://www.annals.org/, and Epidemiology at http://www.epidem.com/). Information on the STROBE Initiative is available at www.strobe-statement.org.
